# Acupoint embedding therapy improves nonalcoholic fatty liver disease with abnormal transaminase: A PRISMA-compliant systematic review and meta-analysis

**DOI:** 10.1097/MD.0000000000018775

**Published:** 2020-01-17

**Authors:** Liang Dai, Vee Voon Ooi, Wenjun Zhou, Guang Ji

**Affiliations:** Institute of Digestive Diseases, Longhua Hospital, Shanghai University of Traditional Chinese Medicine, Shanghai, China.

**Keywords:** acupoint embedding, nonalcoholic fatty liver disease, alanine aminotransferase, systematic review

## Abstract

**Background:**

Non-alcoholic fatty liver disease (NAFLD) with abnormal transaminase were main targeted disorder in clinical intervention. Acupuncture embedding has been used as a modified acupuncture therapy in current management, while no comprehensive summarization has been established. Hence, we conducted a systematic review and meta-analysis to evaluate the effectiveness and safety of acupoint embedding alone or in combination for NAFLD with abnormal transaminase, and to provide potential regimen for further verification.

**Methods:**

Seven English and Chinese databases were systematically researched from inception to February 28, 2019, including PubMed, Embase, Cochrane Central Register of Controlled Trials (CENTRAL), Chinese biomedical literature database (SinoMed), Chinese National Knowledge Infrastructure (CNKI), Chinese VIP information (VIP), and WanFang database. Academic dissertations were also searched as supplement. The searching terms included “nonalcoholic fatty liver disease,” “acupoint embedding,” “clinical trial,” with their corresponding synonyms. Randomized controlled trials (RCTs) and quasi-RCTs involving acupoint embedding alone or in combination for adult patients with NAFLD with abnormal transaminase were included. The diagnosis of NAFLD should be confirmed by radiologic evidence. Two researchers independently completed predefined data sets extraction and quality assessment. STATA 15.0 was applied to estimate the combined effect presented as odds ratio or mean difference (MD) with a 95% confidence interval (CI). The primary outcome was the change of serum alanine aminotransferase (ALT).

**Results:**

A total of fifteen studies with 1349 patients were included. Meta-analysis reported that acupoint embedding alone or in combination was superior to conventional medications on ALT change (MD: 16.58, 95%CI: [10.42, 22.74], P < .001). The benefits were also demonstrated in other outcomes, including aspartate aminotransferase, triglyceride, and total cholesterol, total efficacy rate and radiological efficacy rate. The safety profile of acupoint embedding was satisfactory. BL18 (Ganshu) was the most frequently utilized acupoint.

**Conclusion:**

To some extent, the systematic review supported the application of acupoint embedding in management of NAFLD, while further high-quality studies should be designed to evaluate the practical effect of acupoint embedding.

## Abstract 2

Supplemental Digital Content is available in the text

## 1 Introduction

Nonalcoholic fatty liver disease (NAFLD), as the most common chronic liver disease, affects more than a quarter of adult population.^[1]^ The disease progression of NAFLD is relatively slow; however, once nonalcoholic steatohepatitis (NASH) was confirmed, the risk of cirrhosis and other complications are largely elevated.^[1–4]^ Therefore, preventing NASH progression is a vital step in NAFLD management. Biopsy is the golden standard to diagnose NASH, but performing a biopsy for every patient with NAFLD is not practical due to its invasion, high expenditure, and possible adverse events. Surrogate biochemical indexes like aminotransferases, in particular alanine aminotransferase (ALT), are more frequently used standard to determine the start of treatment, as they were associated with NASH and advanced fibrosis and easy to obtain.^[5]^ Hence, patients with NAFLD with abnormal transaminase were main targeted population in clinical intervention.

Insulin resistance (IR) is one of vital pathologic manifestation in NAFLD progression.^[6]^ Hence, current conventional medications toward NAFLD includes insulin sensitizers, vitamin E, polyene phosphatidyl choline, silymarin, and so on.^[7–9]^ However, none of these agents was officially approved for NAFLD.^[7–9]^ Consequently, many clinicians and patients would consider complementary and alternative interventions, in which acupuncture is one of the main therapies. Various studies have reported that acupuncture could improve IR.^[10,11]^ Recent systematic review reported that acupuncture could significantly reduce the level of homeostasis model assessment for IR by approximate 0.7 in IR-related diseases. These results may indicate acupuncture could benefit the management of NAFLD.

Acupoint embedding is a modified technique based on traditional acupuncture intervention. The nature of this technique is to leave catgut or other absorbable suture threads into the acupoints, therefore to extend stimulation time and further enhance efficacy. This hypothesis has been demonstrated in obesity,^[12]^ which is a common comorbidity of NAFLD and also considered to be closely related to IR. Indeed, a plenty of clinical studies have been conducted to testify the efficacy and safety of acupoint embedding therapy for NAFLD. And some of them did achieve positive results. However, the poor quality of study design and diversity of applied acupoints limit the clinical significance. A rigorous randomized controlled trial (RCT) is definitely needed to confirm the benefit of acupoint embedding and provide standard treatment protocol. Nevertheless, before conducting RCTs, it would be better to comprehensively summarize the available evidences and sort out potential therapeutic protocol for future verification. Therefore, we conducted a systematic review to assess the potential benefits of acupoint embedding alone or in combination by using objective outcome variables, then aimed to provide possible solutions for further validation.

## 2 Methods

### 2.1 Protocol and registration

The protocol was registered with an identification number CRD42019129552 in the PROSPERO database. The systematic review was designed and completed according to Preferred Reporting Items for Systematic Reviews and Meta-Analyses (PRISMA) statement.^[13]^ The corresponding checklist was shown in Table, Supplemental Digital Content. This study was conducted based on available data in various public databases; hence, ethical approval was not necessary.

### 2.2 Literature searching strategy

The clinical studies were retrieved from the authoritative English and Chinese based databases which including PubMed, Embase, Cochrane Central Register of Controlled Trials (CENTRAL), Chinese biomedical literature database (SinoMed), Chinese National Knowledge Infrastructure (CNKI), Chinese VIP information (VIP), and WanFang database. Academic dissertations were not excluded during literature searching. Literature searches were conducted from their inception till February 28, 2019. The searching keywords included “nonalcoholic fatty liver disease,” “acupoint embedding,” “clinical trial,” with their corresponding synonyms as well as for subject terms searching. Detailed searching strategy of every database could be found in File, Supplemental Digital Content.

### 2.3 Study selection and data extraction

We included RCTs and quasi-RCTs to evaluate the efficacy and safety published in both English and Chinese. The qualified participants were defined as age ≥18 years old and diagnosed NAFLD on the foundation of radiologic and/or histologic examinations with abnormal liver aminotransferases. The eligible experimental treatment contained acupoint embedding therapy alone or acupoint embedding therapy in combination with conventional medications or Chinese herbal medicine (CHM). And the comparator should be conventional medications or conventional acupuncture techniques. Exclusion criteria included patients combined with other reason which would induce liver steatosis; nonclinical studies such as animal study; secondary articles such as review and commentary; comparator contained any external interventions, which may interfere acupoint embedding efficacy.

In consideration of providing minimal subjective results, the primary outcome was set to be the change of serum ALT level. Other secondary outcomes included the change of serum aspartate aminotransferase (AST), triglyceride (TG), and total cholesterol (TC), total efficacy rate, radiologic efficacy rate, and adverse events. Due to the certain difference of evaluation criteria in included studies, the evaluation of total efficacy rate was standardized. We defined any improvements in symptoms, biochemical indexes or radiologic examinations as clinical effective. Similarly, radiologic effective was defined as any improvements in any imaging tests.

Two authors (LD and VVO) independently conducted the literature searching, study selection, and data extraction independently. The extracted information consisted of authors and title of study, year of publication, study size, age and gender of the participants, details of methodologic information, details of acupoints, CHM, and western medications used in combination, treatment regimens, details of the control interventions, outcomes, and adverse events. A 3rd individual (GJ) would be introduced to deal with disagreement discussion and consensus establishment.

### 2.4 Quality assessment

The quality of included studies was evaluated by the risk of bias tool based on the Cochrane Handbook of Systematic Reviews of Interventions.^[14]^ Five dimensions were assessed, namely random sequence generation, allocation concealment, blinding method, incomplete outcome data, and selective reporting. Two authors (LD and VVO) independently appraised the risk of bias and disagreement would be addressed by further discussion with a 3rd individual (GJ).

### 2.5 Data analysis

Descriptive analysis was conducted to summarize the findings of participants characteristics and intervention details of the included studies. Two comparisons were arranged, namely acupoint embedding alone or in combination vs conventional medications, and acupoint embedding alone vs electroacupuncture. Data were synthesized by using mean difference (MD) with 95% confidence intervals (CIs) for continuous outcomes (ALT, AST, TG, and TC) or odds ratio (OR) with 95% CI for binary outcomes (total efficacy rate and radiologic efficacy rate) using STATA 15.0 software. The data synthesis model, namely fix-effects or random-effects, was determined based on the heterogeneity. We defined that an *I*^2^ value above 50% indicating substantial heterogeneity. If certain heterogeneity exists, meta-regression would be used to explore the source of heterogeneity, and preset covariates contain participants’ age, baseline data, interventions, and course times. Subgroup analyses were set based on acupoint embedding administration, namely alone, combined with CHM, combined with conventional agents, or combined both CMH and conventional agents. We also performed sensitivity analysis to deal with heterogeneity and testify the stability of results, including comparing effects from different models, influence analysis, and trim and fill test. Publication bias was assessed as well.

## 3 Results

### 3.1 Characteristics of included studies

**Figure 1:**
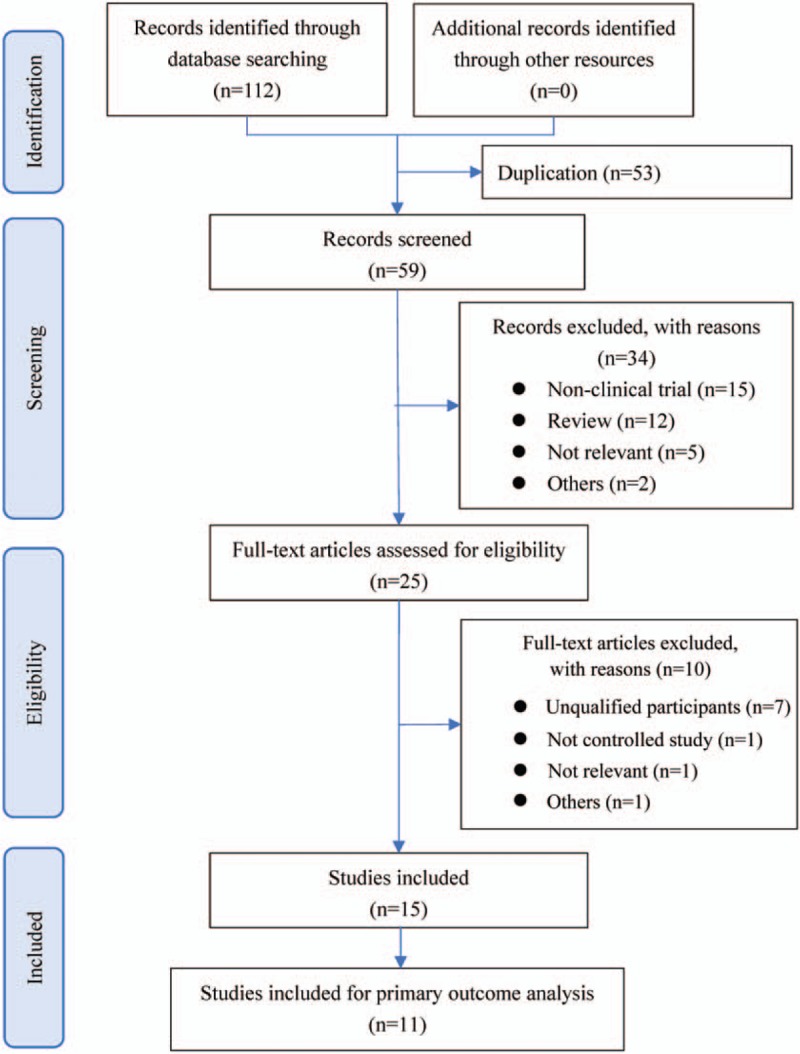
Flowchart of search process and literature selection.

A total of 112 records were obtained from the databases initially, then 59 studies were screening after omitting the duplications. Most citations were excluded due to various reasons. Finally, 15 records were included,^[15–29]^ and 11 records were included for primary outcome analysis.^[15–19,21,24–28]^ The flowchart of search process and literature selection is shown in Figure 1.

All included trials were conducted in China and published in Chinese. A total of 1349 patients with NAFLD were included, with the sample sizes ranged from 50 to 200. The diagnosis of NAFLD was based on official clinical practice guidelines or handbooks, hence all included participants possessed with radiologic evidence of NAFLD. However, no study reported sample size calculation. Common combined diseases contained obesity, diabetes, and hyperlipidemia. Three studies utilized electroacupuncture as comparator, and the rest used conventional medications. The detailed characteristics of included studies are presented in Table 1 .

**Table 1:**
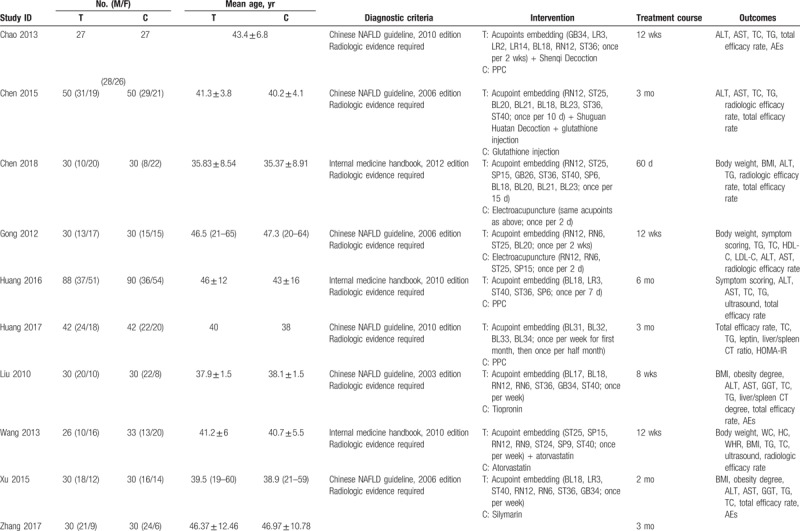
Characteristics of included studies.

**Table 1 (Continued):**
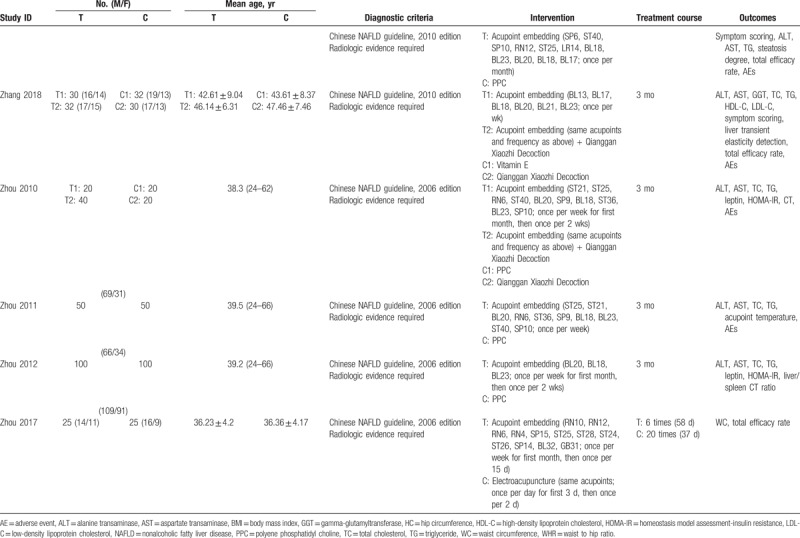
Characteristics of included studies.

### 3.2 Serum ALT change

**Figure 2:**
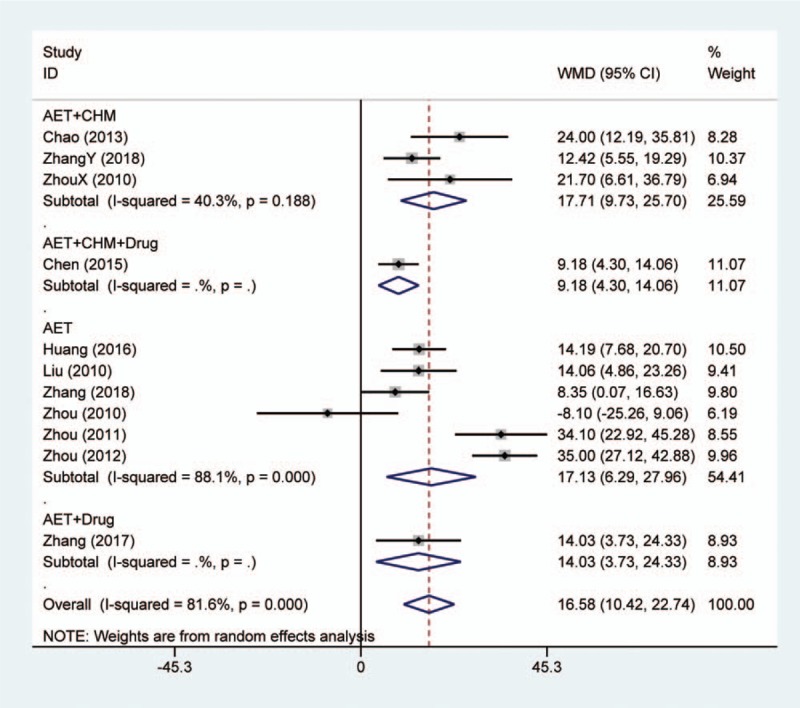
Forest plot of serum alanine aminotransferase change in comparison of acupoint embedding alone or in combination vs conventional medications. AET = acupoint embedding therapy, CHM = Chinese Herbal Medicine, CI = confidence interval.

Nine studies using conventional medications as comparator reported the change of serum ALT level. Meta-analysis showed that acupoint embedding alone or in combination could bring more reduction in ALT level than conventional medications (MD: 16.58, 95% CI: [10.42, 22.74], *P* < .001) (Fig. 2). The heterogeneity test showed *I*^2^ = 81.6% and *P* < .001. For the subgroup analysis, 6 studies compared acupoint embedding alone with conventional agents, the synthesized result was similar to overall effect (MD: 17.13, 95% CI: [6.29, 27.96], *P* = .002). Three studies reported the effect size of acupoint embedding combined with CHM vs conventional drugs, and the result was also consistent (MD: 17.71, 95% CI: [9.73, 25.70], *P* < .001). Only 1 study evaluated the add-on effect of acupoint embedding based on conventional medications, which was reported as (MD: 14.03, 95% CI: [3.73, 24.33], *P* = .008). Besides, 1 study used most complicated interventions, namely acupoint embedding combined with CHM and conventional medication, and the result showed better improvement in treatment group as well.

Among 3 studies using electroacupuncture as comparator, only 2 reported serum ALT level. The synthesized result did not indicate any efficacy difference between acupoint embedding and electroacupuncture (MD: 0.84, 95% CI: [2.98, 4.65], *P* = .668) (Fig. 1, Supplemental Digital Content).

### 3.3 Secondary outcomes

**Figure 3:**
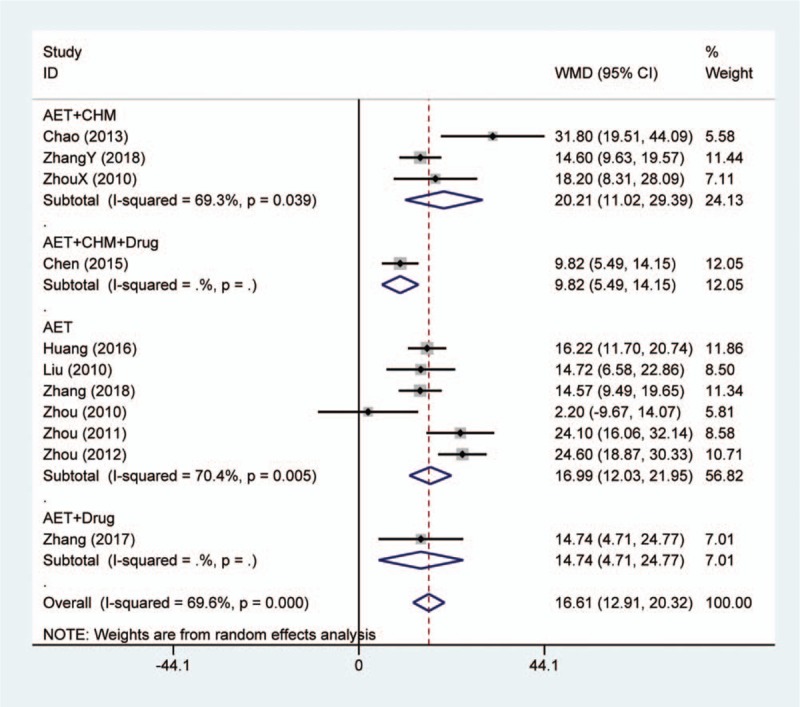
Forest plot of serum aspartate aminotransferase change in comparison of acupoint embedding alone or in combination vs conventional medications. AET = acupoint embedding therapy, CHM = Chinese Herbal Medicine, CI = confidence interval.

Ten studies reported the change of serum AST level. For conventional medications as comparator, the synthesized result of 9 studies showed that acupoint embedding alone or in combination were superior in reducing AST (MD: 16.61, 95% CI: [12.91, 20.32], *P* < .001) (Fig. 3). The heterogeneity test showed *I*^2^ = 69.6% and *P* < .001. Subgroup analysis did not indicate any variation among different treatment strategies. Besides, 1 study compared acupoint embedding with electroacupuncture in AST improvement, and there was no significance between these 2 interventions.^[18]^

**Figure 4:**
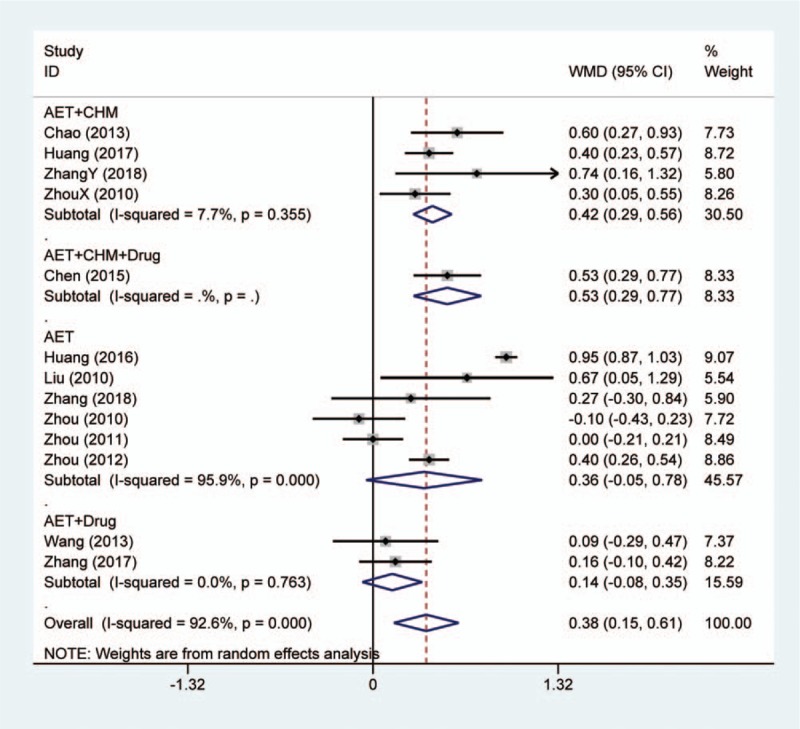
Forest plot of serum triglyceride change in comparison of acupoint embedding alone or in combination vs conventional medications. AET = acupoint embedding therapy, CHM = Chinese Herbal Medicine, CI = confidence interval.

Among the included studies, thirteen studies reported the change of serum TG after treatment. For conventional medicines as comparator, meta-analysis indicated that acupoint embedding alone or in combination had better efficacy in decreasing TG (MD: 0.38, 95% CI: [0.15, 0.61], *P* = .001) (Fig. 4). The heterogeneity test indicated *I*^2^ = 92.6% and *P* < .001. However, the results between different subgroups did not keep consistent. Acupoint embedding combined with CHM showed better efficacy than convention treatment (MD: 0.42, 95% CI: [0.29, 0.56], *P* < .001). Besides, the single study involved both CHM and conventional agents in combination showed that the combination could also obtain larger TG reduction than general intervention.^[16]^ The other 2 categories did not show superior effect. Among 3 studies using electroacupuncture as comparator, 2 reported the change of serum TG level. The synthesized result showed that no meaningful difference between 2 interventions (MD: 0.01, 95% CI: [−0.26, 0.28], *P* = .941) (Fig. 2, Supplemental Digital Content).

**Figure 5:**
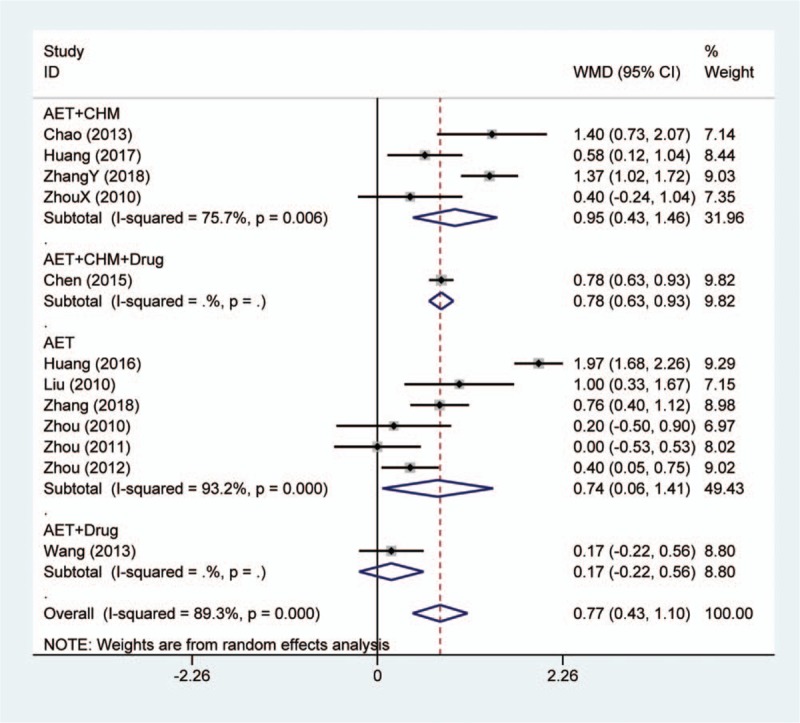
Forest plot of serum total cholesterol change in comparison of acupoint embedding alone or in combination vs conventional medications. AET = acupoint embedding therapy, CHM = Chinese Herbal Medicine, CI = confidence interval.

Change of serum TC was reported in 11 trials. Meta-analysis illustrated that acupoint embedding alone or in combination could reduce TC more than conventional medications (MD: 0.77, 95% CI: [0.43, 1.10], *P* < .001) (Fig. 5). The results of heterogeneity test were *I*^2^ = 89.3% and *P* < .001. Among 4 subgroups, only the single study of acupoint embedding combined with conventional medication revealed no extra benefits from adding acupoint embedding. In addition, 1 study assessed the change of TC by using electroacupuncture as comparator.^[17]^ The result showed no statistical difference between 2 interventions as well.

Total efficacy rate and radiologic efficacy rate were reported in 9 and 4 studies, respectively. After homogenization, meta-analysis was conducted based on available data. Acupoint embedding alone or in combination presented better total efficacy rate than conventional medications (OR: 3.85, 95% CI: [2.50, 5.93], *P* < .001) (Fig. 3, Supplemental Digital Content). The heterogeneity test showed *I*^2^ = 0.0% and *P* = .781. Besides, the synthesized results showed acupoint embedding had better total efficacy rate compared with electroacupuncture (OR: 3.08, 95% CI: [1.10, 8.57], *P* = .032) (Fig. 4, Supplemental Digital Content). For radiologic efficacy rate, 1 study assessed the add-on effect of acupoint embedding based on conventional therapy,^[22]^ 1 study evaluated the triple combination compared with conventional therapy,^[16]^ and the rest 2 appraised the difference between acupoint embedding and electroacupuncture.^[17,18]^ The introduction of acupoint embedding in conventional therapy did not show extra radiologic improvement. Triple combination presented better radiologic efficacy rate than traditional interventions. These results were not considered for synthesis due to obvious difference in experimental interventions. Meta-analysis indicated that no statistical difference between acupoint embedding and electroacupuncture (OR: 0.87, 95% CI: [0.30, 2.48], *P* = .789) (Fig. 5, Supplemental Digital Content).

### 3.4 Adverse events

Seven studies reported the safety profile of interventions. Documented adverse events included painful wound, rashes, subcutaneous nodulations, low fever, and gastrointestinal symptoms. No serious adverse event was reported. All studies claimed that all adverse events were well managed and did not cause significant change in findings and withdrawal. However, due to the limited data, meta-analysis could not be conducted.

### 3.5 Acupoint summarization

**Figure 6:**
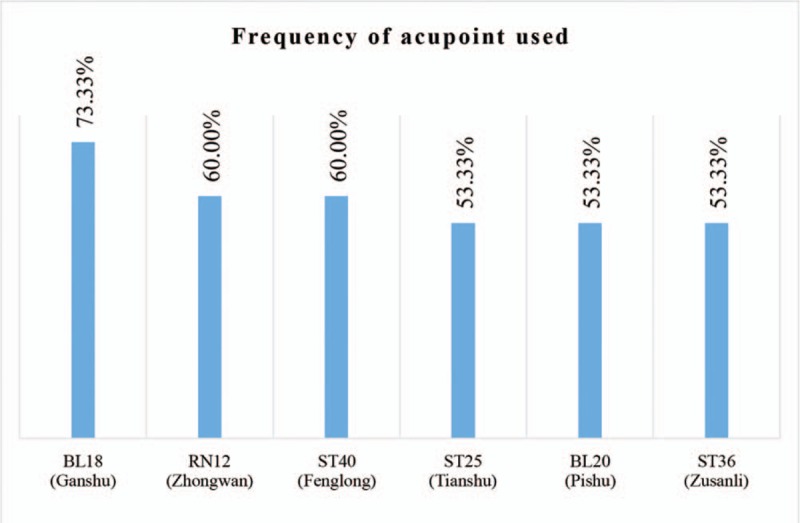
Acupoints used by more than half included studies.

More than 30 acupoints were extracted from included studies. We summarized the commonly used acupoints which were selected by more than half studies (Fig. 6). BL18 (Ganshu) was most utilized, followed by RN12 (Zhongwan) and ST40 (Fenglong).

### 3.6 Methodologic quality

**Figure 7:**
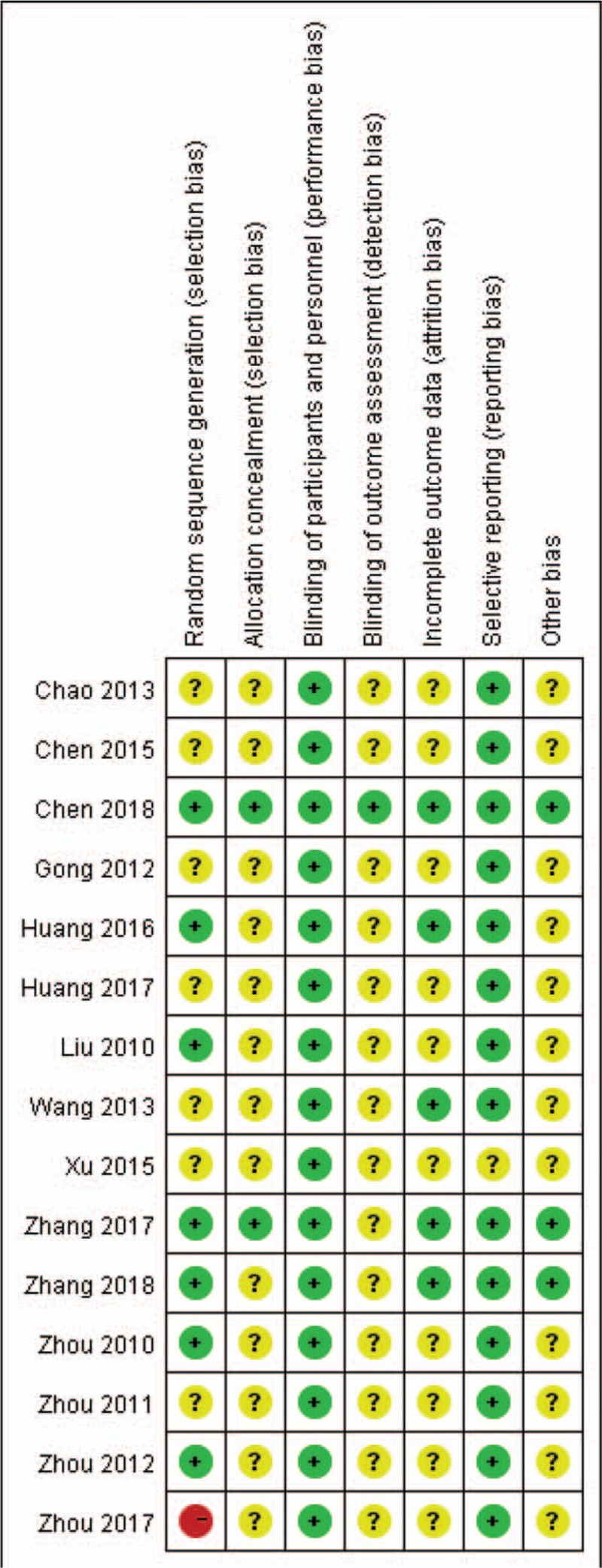
Risk of bias summary: review authors’ judgments about each risk of bias item for each included study.

**Figure 8:**
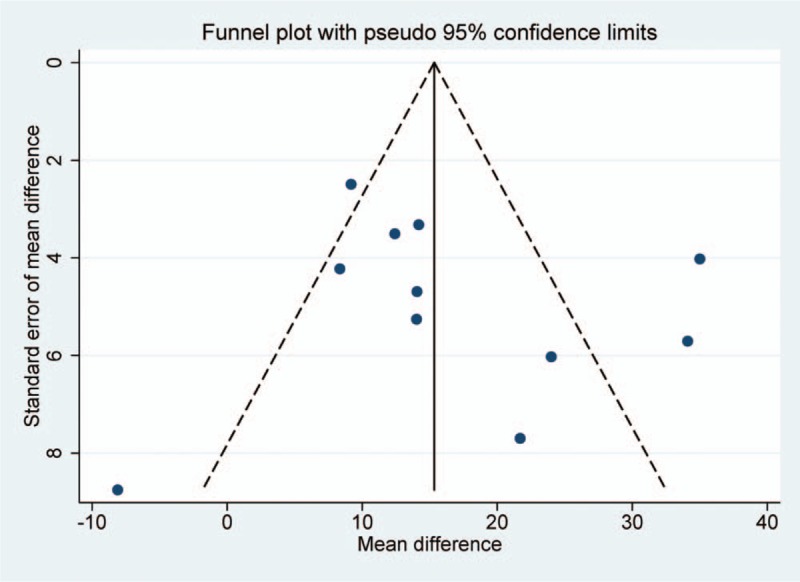
Funnel plot of serum alanine aminotransferase change in comparison of acupoint embedding alone or in combination vs conventional medications.

The evaluation of risk of bias for each study is presented in Figure 7. Most of included studies claimed randomization; however, only 7 specified the method. One study allocated participants according to intervention methods, hence high risk of bias was determined. Only 1 study documented the allocation concealment method. Due to the characteristics of acupoint embedding, blinding of participants and researchers was not realistic for these studies, so that this category was generally considered as low risk. Nevertheless, blinding of assessors was only mentioned in 1 study. Selective reporting bias was found in 1 study as the predetermined outcomes were not recorded. Publication bias was assessed based on primary outcome (Fig. 8). The funnel plot indicated that certain bias existed.

### 3.7 Meta-regression and sensitivity analysis

Both *I*^2^ statistical magnitude and Galbraith radial plot (Fig. 6, Supplemental Digital Content) indicated certain heterogeneity of the results. Hence, meta-regression analysis was conducted based on primary outcome. However, none of the preset covariates, namely participants’ age, baseline serum ALT level, interventions and course times, showed significant association with effect size (Table 2). These results suggested that the heterogeneity may be resulted from inherent attributes of studies.

**Table 2:**
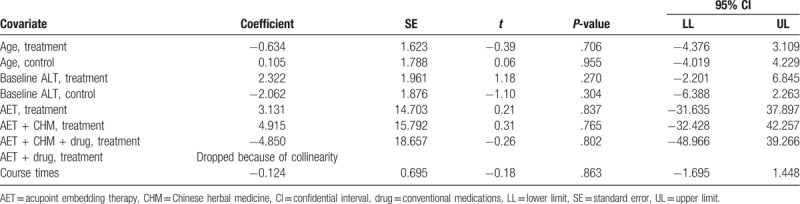
Meta-regression model with univariate analysis of interested covariates.

To deal with heterogeneity and verify the result, sensitivity analyses were conducted based on primary outcome by using different statistical model, influence analysis, and trim and fill test. By using fix-model, the result still showed that acupoint embedding alone or in combination could bring more reduction in ALT level than conventional medications (MD: 15.34, 95% CI: [12.85, 17.83], *P* < .001) (Fig. 7, Supplemental Digital Content). Based on influence analysis, the synthesized effect fluctuated in the 95% CI of original effect by eliminating any one of the included studies (Fig. 8, Supplemental Digital Content). Besides, the trim and fill test did not introduce any hypothetical studies, hence the effect did not change (Fig. 9, Supplemental Digital Content). All these methods indicated that the improvement of ALT presented good stability, though certain heterogeneity and bias exist.

## 4 Discussion

As an idiomatical external intervention of TCM, acupuncture is considered to possess various physiologic functions, including neurohumoral regulation, immunoregulation, lipidolysis effect, and so on. Hence, it has been utilized in multiple system disorders, no exception for NAFLD.^[30,31]^ On the foundation of acupuncture principle, acupoint embedding was developed, aiming to enhance the efficacy and reduce operation times in the meantime.^[32]^ Our result did demonstrated that acupoint embedding alone or in combination showed better effectiveness in treating NAFLD with abnormal transaminase compared with conventional medications. However, the conclusion should be interpreted with caution, as high heterogeneity existed in included studies. Further rigorous RCT is urgent to design. On the foundation of this systematic review, potential therapeutic regimen may be considered as following: BL18 (Ganshu), RN12 (Zhongwan), ST40 (Fenglong), ST25 (Tianshu), BL20 (Pishu), and ST36 (Zusanli) as major acupoints, with at least 3-month treatment course.

Acupoint embedding is a modified technique of acupuncture. By inserting absorbable string into the acupoints, the stimulation time would dramatically increase comparing with traditional acupuncture, and so operation frequencies would reduce.^[32]^ Theoretically, the efficacy brought by acupoint embedding would be better than normal acupuncture, whereas, although only three studies were included, our results did not show the superiority of acupoint embedding to electroacupuncture. Hence, if efficacy is the only concern, acupoint embedding may not be prior choice. When taking operation convenience into account, acupoint embedding has its own advantages.

The NAFLD is a metabolic disorder characterized by abnormal lipid metabolism and IR. The specific pathogenesis is still unknown, but management of NAFLD could not bypass these 2 mechanisms. Previous studies have reported that acupuncture could improve lipid metabolism. To be specific, acupuncture on ST40 (Fenglong) and RN4 (Guanyuan) could increase the activity of lipoprotein lipase and hepatic lipase, then improve liver steatosis in hyperlipidemia rat.^[33]^ Other studies also reported that acupuncture on ST40 (Fenglong), ST36 (Zusanli), LR3 (Taichong), and SP6 (Sanyinjiao) could regulate related hormones like leptin and adiponectin.^[34,35]^ It was illustrated that possible signal pathways included serum retinol-binding protein 4, liver X receptor α, sterol-regulatory element binding protein-1c and peroxisome proliferator-activated receptor-α.^[36–38]^ Our meta-analysis showed that the lipid-lowering effect of acupoint embedding in NAFLD is definite, which is in accordance with previous findings. In addition, 3 included studies evaluated the change of leptin. Acupoint embedding alone or in combination with CHM could significant decrease the level of serum leptin.^[20,27,28]^

Currently, IR is a confirmed factor in NAFLD pathogenesis. Recent studies demonstrated that acupuncture on ST40 (Fenglong), ST36 (Zusanli), and LR3 (Taichong) could significantly reduce homeostasis model assessment of IR index and improve glucose metabolism.^[39,40]^ Moreover, several studies reported that acupuncture could alleviate the oxidative stress and inflammatory factor overexpression,^[41,42]^ which may be induced by IR. The relevant acupoints were similar as previous, including ST40 (Fenglong), ST36 (Zusanli), LR3 (Taichong), and SP6 (Sanyinjiao). Although we did not conduct meta-analysis due to limited data, the available results from included studies were consistent in improving IR.^[20,27,28]^

Plenty of systematic reviews from acupuncture area have been published to summarize the available evidences. However, none of them would not face the challenge brought by quality of included studies.^[43,44]^ Our review is not an exception. Due to the specialty of acupoint embedding, unable blinding of participants and clinicians could be understood. However, other dimensions also did not show satisfactory responses. Unclear risk of bias is the commonest evaluation due to the unobtainable of study protocol and unclear description of study reports. The searching in World Health Organization International Clinical Trials Registry Platform (WHO-ICTRP) also came back with disappointed results. Therefore, it is urgent to educate researchers the whole view of clinical study, namely from protocol design, to trial registration, then to result reporting, and the international standard guidelines should be utilized as primary tools.^[45–47]^

Our study has certain limitations. Firstly, baseline difference among included studies was omitted. Based on the description of included studies, the range of ALT level was relatively broad, which may introduce bias in the synthesized results. Although the meta-regression did not demonstrate that baseline differences induced the heterogeneity. Secondly, although we used the objective change of serum ALT level as primary outcome, this outcome could not fully explain the efficacy. In general, relative clinical studies would use patients with 30% reduction or restoration to normal as their primary variable. But the data from included studies could not support this analysis. Moreover, though every included study required radiologic evidence to include participants, the extracted data did not involve standard qualitative or quantitative radiologic assessments of NAFLD. Four studies did document the results of imaging tests; however, every study utilized individual system to evaluate the improvement. This made our synthesis should go through homogenization, which may bring further bias.

## 5 Conclusion

In summary, the systematic review partially demonstrated the reasonability of acupoint embedding in management of NAFLD, while more consolidated evidences are still needed. Further high-quality clinical studies should be design to explain the actual effectiveness of acupoint embedding using suitable sham control. The other focus could be the effect brought by the combination with conventional medications due to limited data available.

## Author contributions

**Conceptualization:** Liang Dai.

**Data curation:** Vee Voon Ooi.

**Formal analysis:** Liang Dai, Vee Voon Ooi.

**Funding acquisition:** Guang Ji.

**Investigation:** Liang Dai, Vee Voon Ooi.

**Methodology:** Liang Dai.

**Project administration:** Liang Dai, Vee Voon Ooi, Wenjun Zhou.

**Resources:** Liang Dai, Vee Voon Ooi.

**Software:** Liang Dai.

**Supervision:** Wenjun Zhou, Guang Ji.

**Validation:** Wenjun Zhou, Guang Ji.

**Visualization:** Liang Dai.

**Writing – original draft:** Liang Dai, Vee Voon Ooi.

**Writing – review & editing:** Wenjun Zhou, Guang Ji.

Guang Ji orcid: 0000-0003-0842-3676.

## Supplementary Material

Supplemental Digital Content

## Supplementary Material

Supplemental Digital Content

## Supplementary Material

Supplemental Digital Content

## Supplementary Material

Supplemental Digital Content

## Supplementary Material

Supplemental Digital Content

## Supplementary Material

Supplemental Digital Content

## Supplementary Material

Supplemental Digital Content

## Supplementary Material

Supplemental Digital Content

## Supplementary Material

Supplemental Digital Content

## Supplementary Material

Supplemental Digital Content

## Supplementary Material

Supplemental Digital Content
