# Supplementary material for: Acupoint embedding therapy improves nonalcoholic fatty liver disease with abnormal transaminase: A PRISMA-compliant systematic review and meta-analysis
Source: Medicine (Baltimore). 2020 Jan 17;99(3):e18775. doi: 10.1097/MD.0000000000018775 (PMC7220490; doi:10.1097/MD.0000000000018775)
Supplement: Supplemental Digital Content [file medi-99-e18775-s002.docx]

File, Supplemental Digital Content. Electronic search strategy

- **English**

PUBMED

((((((((Non-alcoholic Fatty Liver Disease[MeSH Terms]) OR non-alcoholic fatty liver disease) OR non-alcoholic steatohepatitis) OR non-alcoholic fatty liver) OR non-alcoholic cirrhosis))) AND (((((((((acupoint embedding) OR thread embedding) OR catgut embedding) OR thread inserting) OR thread injecting) OR embedding acupuncture) OR catgut inserting) OR catgut injecting) OR acupuncture therapy[MeSH Terms])) AND (((((clinical study) OR clinical trial) OR clinical observation) OR control study) OR Epidemiologic Study Characteristics[MeSH Terms])

CENTRAL

ID Search

#1 ("non-alcoholic fatty liver disease" OR "non-alcoholic steatohepatitis" OR "non-alcoholic fatty liver" OR "non-alcoholic cirrhosis"):ti,ab,kw (Word variations have been searched)

#2 MeSH descriptor: [Non-alcoholic Fatty Liver Disease] explode all trees

#3 "acupoint embedding" OR "thread embedding" OR "catgut embedding" OR "thread inserting" OR "thread injecting" OR "embedding acupuncture" OR "catgut inserting" OR "catgut injecting"

#4 MeSH descriptor: [Acupuncture Therapy] explode all trees

#5 "clinical study" OR "clinical trial" OR "clinical observation" OR "control study"

#6 MeSH descriptor: [Epidemiologic Study Characteristics] explode all trees

#7 #1 OR #2

#8 #3 OR #4

#9 #5 OR #6

#10 #7 AND #8 AND #9

EMBASE

('non-alcoholic fatty liver disease'/exp OR 'non-alcoholic fatty liver disease' OR 'non-alcoholic steatohepatitis'/exp OR 'non-alcoholic steatohepatitis' OR 'non-alcoholic fatty liver'/exp OR 'non-alcoholic fatty liver' OR 'non-alcoholic cirrhosis' OR 'nonalcoholic fatty liver'/exp) AND ('acupoint embedding' OR 'thread embedding' OR 'catgut embedding'/exp OR 'catgut embedding' OR 'thread inserting' OR 'thread injecting' OR 'embedding acupuncture' OR 'catgut inserting' OR 'catgut injecting' OR 'acupuncture'/exp) AND ('clinical study' OR 'clinical trial'/exp OR 'clinical trial' OR 'clinical observation'/exp OR 'clinical observation' OR 'control study' OR 'clinical study'/exp)

- **Chinese**

CNKI

SU=('非酒精性脂肪性肝病'+'单纯性脂肪肝'+'非酒精性脂肪肝'+'非酒精性脂肪肝炎'+'非酒精性肝硬化') AND SU=('穴位埋线'+'埋线'+'埋藏疗法'+'羊肠线治疗'+'埋线治疗'+'埋线疗法'+'埋藏治疗'+'穴位埋线疗法'+'穴位埋线法') AND SU=('随机'+'对照'+'临床试验'+'临床观察'+'临床研究'+'临床治疗'+'疗效观察')

Wanfang

主题:(非酒精性脂肪性肝病+非酒精性脂肪性肝炎+非酒精性脂肪性肝+非酒精性肝硬化+单纯性脂肪肝)*主题:(随机+对照+临床试验+临床观察+临床研究+临床治疗+疗效观察)*主题:(穴位埋线+埋线+埋藏疗法+穴位埋线法+羊肠线疗法+埋线治疗+埋线疗法)

VIP

M=(非酒精性脂肪性肝病 OR 单纯性脂肪肝 OR 非酒精性脂肪肝 OR 非酒精性脂肪肝炎 OR 非酒精性肝硬化) AND M=(穴位埋线 OR 埋线 OR 埋藏疗法 OR 羊肠线治疗 OR 埋线治疗 OR 埋线疗法 OR 埋藏治疗 OR 穴位埋线疗法 OR 穴位埋线法) AND M=(随机 OR 对照 OR 临床试验 OR 临床观察 OR 临床研究 OR 临床治疗 OR 疗效观察)

Sinomed

27 (#26) AND (#17) AND (#6)

26 (#25) OR (#24) OR (#23) OR (#22) OR (#21) OR (#20) OR (#19) OR (#18)

25 "治疗性人类试验"[不加权:扩展]

24 "疗效观察"[常用字段:智能]

23 "临床治疗"[常用字段:智能]

22 "临床研究"[常用字段:智能]

21 "临床观察"[常用字段:智能]

20 "临床试验"[常用字段:智能]

19 "对照"[常用字段:智能]

18 "随机"[常用字段:智能]

17 (#16) OR (#15) OR (#14) OR (#13) OR (#12) OR (#11) OR (#10) OR (#9) OR (#8) OR (#7)

16 "穴位结扎"[不加权:扩展]

15 "穴位埋线法"[常用字段:智能]

14 "穴位埋线疗法"[常用字段:智能]

13 "埋藏治疗"[常用字段:智能]

12 "埋线疗法"[常用字段:智能]

11 "埋线治疗"[常用字段:智能]

10 "羊肠线治疗"[常用字段:智能]

9 "埋藏疗法"[常用字段:智能]

8 "埋线"[常用字段:智能]

7 "穴位埋线"[常用字段:智能]

6 (#5) OR (#4) OR (#3) OR (#2) OR (#1)

5 "非酒精性脂肪性肝病"[常用字段:智能]

4 "非酒精性肝硬化"[常用字段:智能]

3 "非酒精性脂肪肝炎"[常用字段:智能]

2 "非酒精性脂肪肝"[常用字段:智能]

1 "单纯性脂肪肝"[常用字段:智能]
