# Supplementary material for: Acupoint embedding therapy improves nonalcoholic fatty liver disease with abnormal transaminase: A PRISMA-compliant systematic review and meta-analysis
Source: Medicine (Baltimore). 2020 Jan 17;99(3):e18775. doi: 10.1097/MD.0000000000018775 (PMC7220490; doi:10.1097/MD.0000000000018775)
Supplement: Supplemental Digital Content [file medi-99-e18775-s010.pdf]

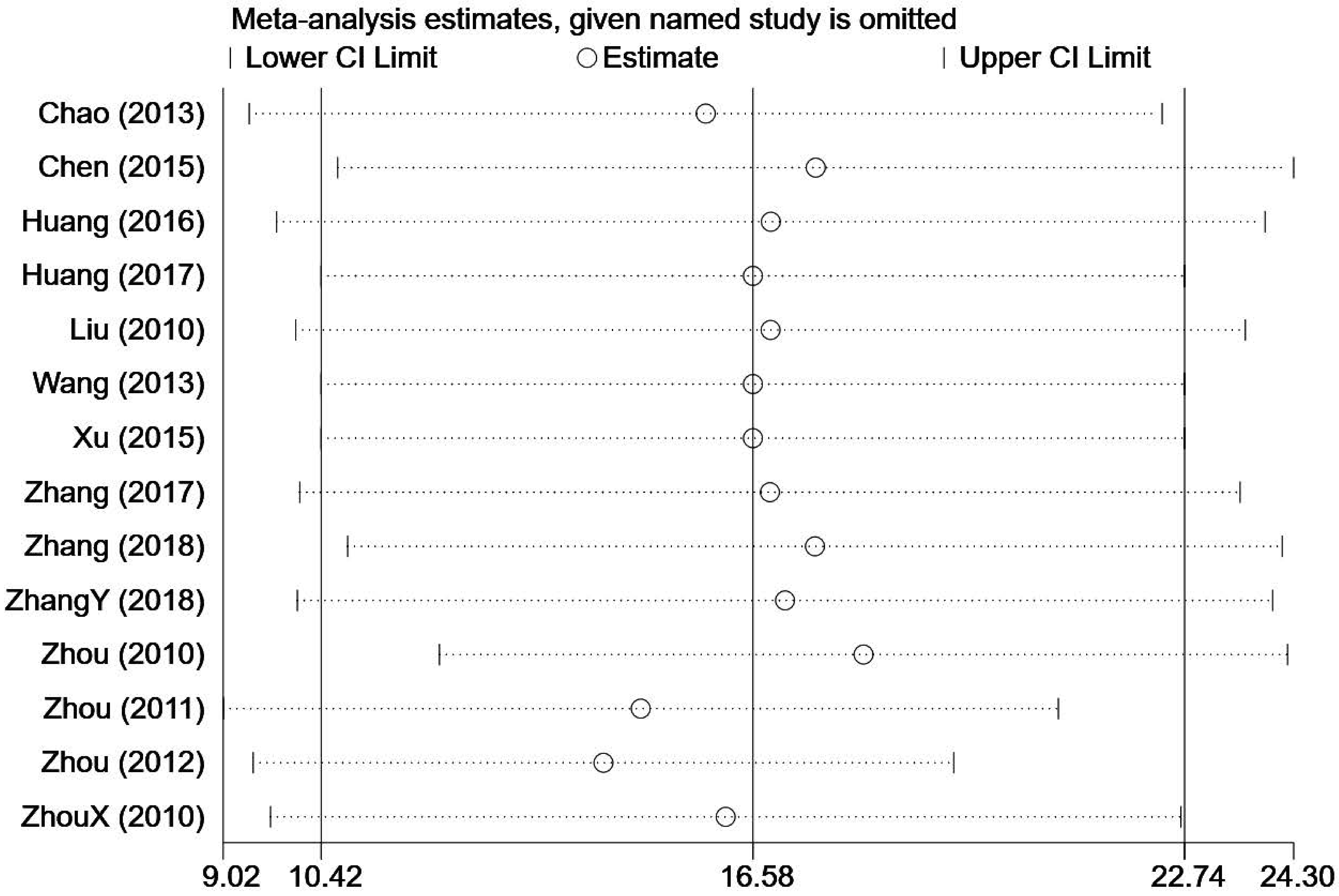

Fig. 8, Supplemental Digital Content. Influence analysis of serum ALT change in comparison of acupoint embedding alone or in combination versus conventional medications
